# Supplementary material for: DMRforPairs: identifying Differentially Methylated Regions between unique samples using array based methylation profiles
Source: BMC Bioinformatics. 2014 May 15;15:141. doi: 10.1186/1471-2105-15-141 (PMC4046028; doi:10.1186/1471-2105-15-141)
Supplement: Additional file 2 — DMRforPairs output for the comparison of A431-MCF7 and NA17018-NA17105. Please start from the HTML files in each folder. Available via the BMC Bioinformatics website. [file 1471-2105-15-141-S2.zip › 1394847754114233_MOESM2_ESM/A431_MCF7/figures/15286.pdf]

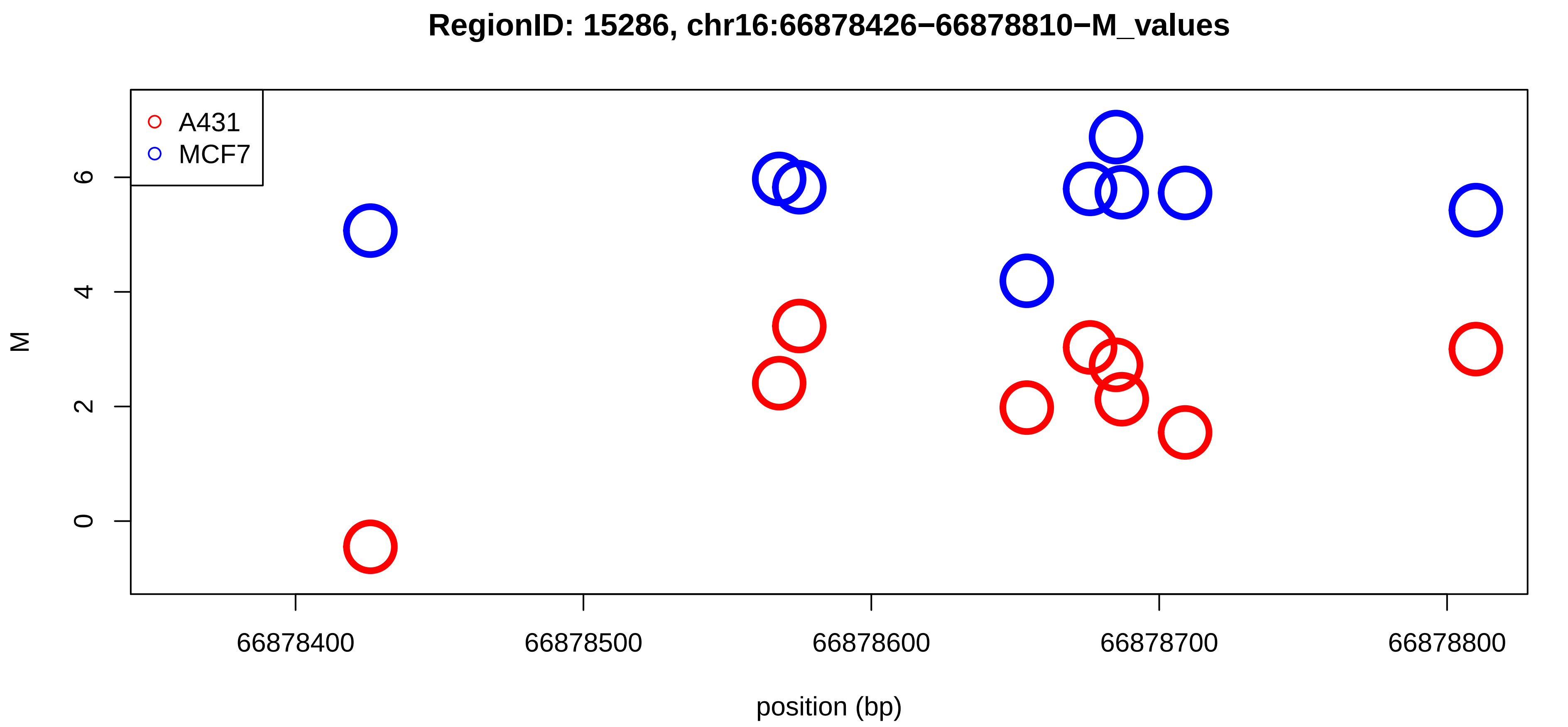
RegionID: 15286, chr16:66878426−66878810−M_values
A431
MCF7
M
0
2
4
6
66878400
66878500
66878600
66878700
66878800
position (bp)

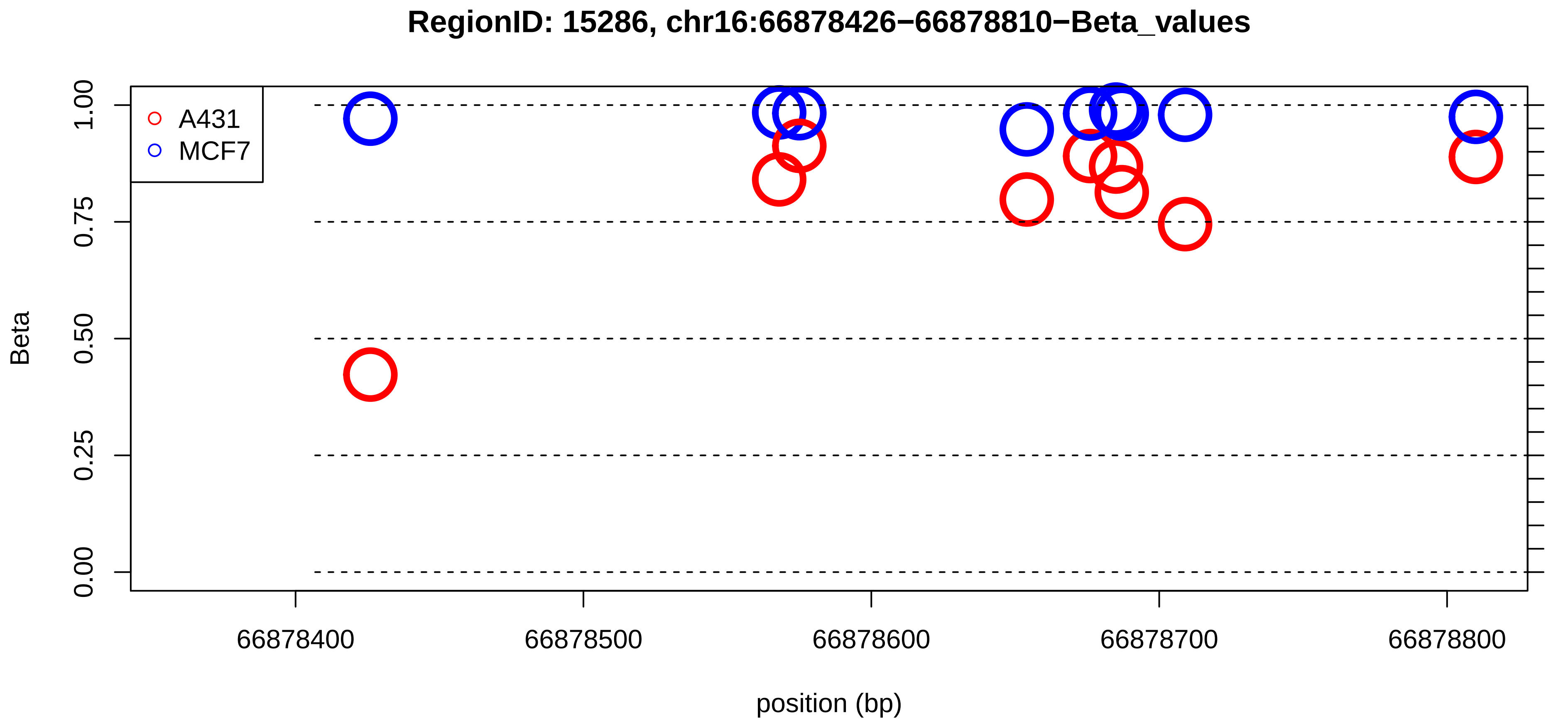
RegionID: 15286, chr16:66878426−66878810−Beta_values
A431
MCF7
Beta
0.00
0.25
0.50
0.75
1.00
66878400
66878500
66878600
66878700
66878800
position (bp)

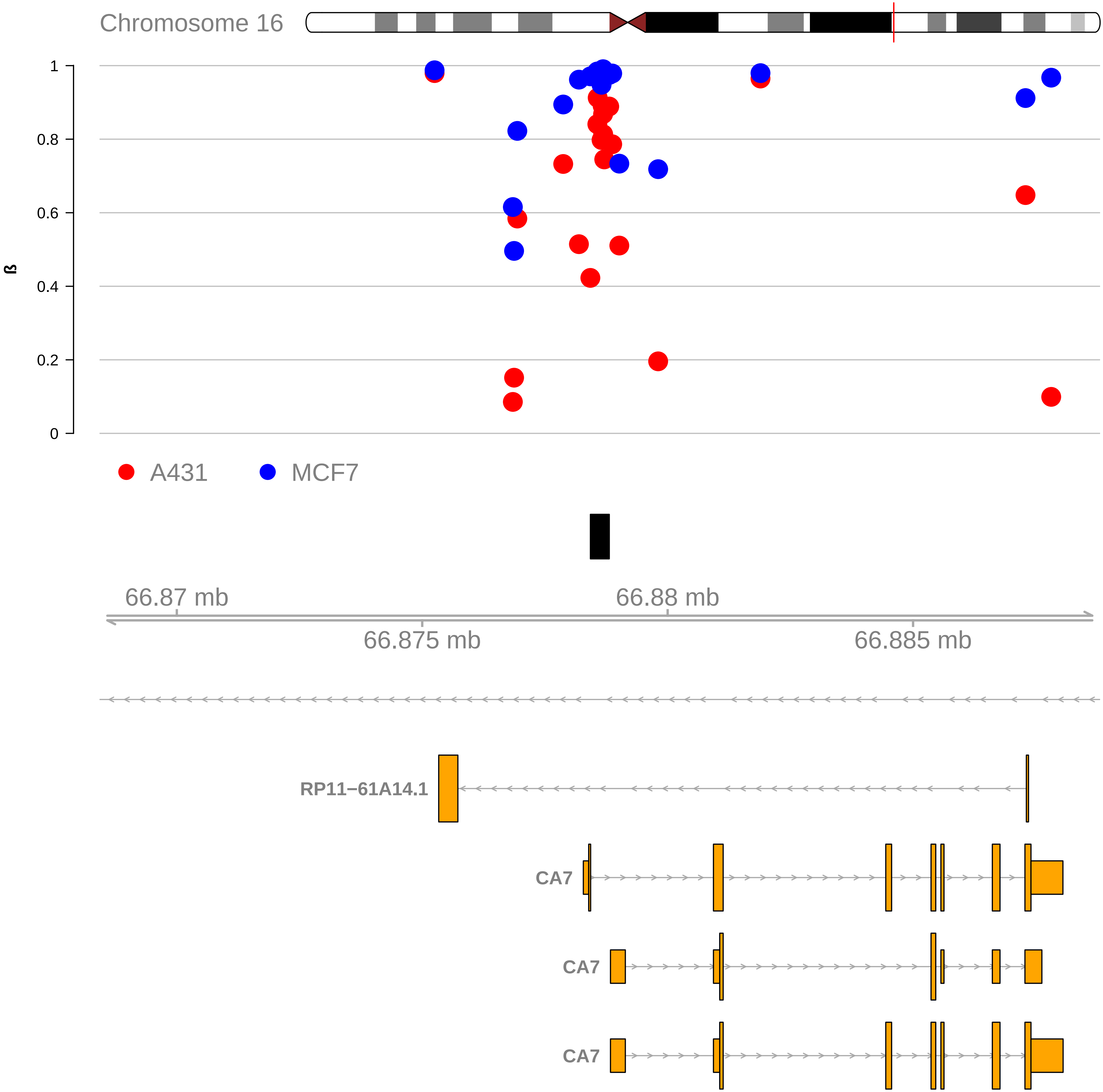

Chromosome 16
1
0.8
0.6
0.4
0.2
0
ß
A431
MCF7
66.87 mb
66.88 mb
66.875 mb
66.885 mb
RP11−61A14.1
CA7
CA7
CA7
